# Factors Associated with Early Introduction of Formula and/or Solid, Semi-Solid or Soft Foods in Seven Francophone West African Countries

**DOI:** 10.3390/nu7020948

**Published:** 2015-01-30

**Authors:** Abukari I. Issaka, Kingsley E. Agho, Andrew N. Page, Penelope L. Burns, Garry J. Stevens, Michael J. Dibley

**Affiliations:** 1 School of Medicine, University of Western Sydney, Penrith NSW 2751, Australia; E-Mails: p.burns@uws.edu.au (P.L.B.); G.Stevens@uws.edu.au (G.J.S.); 2 School of Science and Health, University of Western Sydney, Penrith NSW 2751, Australia; E-Mails: k.agho@uws.edu.au (K.E.A.); a.page@uws.edu.au (A.N.P.); 3 Sydney School of Public Health, Edward Ford Building (A27), University of Sydney, NSW 2006, Australia; E-Mail: michael.dibley@sydney.edu.au

**Keywords:** solid, semi-solid or soft foods, breastfeeding, francophone, West Africa

## Abstract

The aim of this study was to identify factors associated with early introduction of formula and/or solid, semi-solid or soft foods to infants aged three to five months in seven Francophone West African countries. The sources of data for the analyses were the most recent Demographic and Health Survey datasets of the seven countries, namely Benin (BDHS, 2012), Burkina Faso (BFDHS, 2010), Cote d’Ivoire (CIDHS, 2011–2012), Guinea (GDHS, 2012), Mali (MDHS, 2012–2013), Niger (NDHS, 2012) and Senegal (SDHS, 2010). The study used multiple logistic regression methods to analyse the factors associated with early introduction of complementary feeding using individual-, household- and community-level determinants. The sample was composed of 4158 infants aged between three and five months with: 671 from Benin, 811 from Burkina Faso, 362 from Cote d’Ivoire, 398 from Guinea, 519 from Mali, 767 from Niger and 630 from Senegal. Multiple analyses indicated that in three of the seven countries (Benin, Guinea and Senegal), infants who suffered illnesses, such as diarrhoea and acute respiratory infection, were significantly more likely to be introduced to formula and/or solid, semi-solid or soft foods between the age of three and five months. Other significant factors included infants who: were born in second to fourth position (Benin), whose mothers did not attend any antenatal clinics (Burkina Faso and Niger), were male (Cote d’Ivoire and Senegal), lived in an urban areas (Senegal), or were delivered by traditional birth attendants (Guinea, Niger and Senegal). Programmes to discourage early introduction of formula and/or solid, semi-solid or soft foods in these countries should target the most vulnerable segments of the population in order to improve exclusive breastfeeding practices and reduce infant mortality.

## 1. Introduction

There is extensive evidence in the extant literature on the benefits of exclusive breastfeeding (EBF) and risks of infant morbidity and mortality posed by non-EBF [1,2,3]. Due to its importance, there has been a global recommendation [4] for EBF to be promoted in infants and young children for the first six months of life, with continued breastfeeding for up to two years or beyond. The practice of EBF entails giving the infant breast milk only, with the exception of medications, such as oral dehydration salts, syrups, minerals and vitamins. Non-EBF during the first six months of life has been found to result in 1.4 million deaths and contribute to 10% of disease burden in children younger than five years [5]. Despite its benefits, in most West African countries, a relatively small proportion of children under six months of age are exclusively breastfed [6].

One practice that results in non-EBF is the early introduction of formula and/or solid, semi-solid or soft foods to infants aged between three and five months. Introduction of formula and/or solid, semi-solid or soft foods is defined as “the proportion of children aged 6–8 months who receive solid, semi-solid or soft foods” [7]. Early (before six months) introduction of formula and/or solid, semi-solid or soft foods can be disadvantageous for the infant. It can replace nutrient-dense breast milk or formula to result in inadequate nutrients and energy for growth [8]. Extant paediatric literature is replete with studies that have highlighted the association of early introduction of complementary feeding with poor nutritional status, diarrhoea and respiratory infections in infants [9,10,11]. The existing literature suggests that there is an increased risk of developing diarrheal illnesses in infants who are not exclusively breastfed in the early months of life. According to a past UNICEF report [12], despite the fact that breastfeeding is a universal practice in West Africa, with a mean duration of 20 months, the rate of EBF is lower than in any other region of the world.

In a recent report [13], the latest EBF rates for the seven countries under examination were: 33% (Benin 2012), 24.8% (Burkina Faso 2010), 12.1% (Cote d’Ivoire 2012), 21% (Guinea 2012), 35% (Mali 2013), 23% (Niger 2012) and 38% (Senegal 2012). These low rates of EBF may suggest that infants in these countries were more prone to diarrheal diseases as a result of consuming contaminated or unwholesome foods or water, which is reflected in the distribution of the causes of death among children less than five years due to diarrhoea, in these countries: 7.7% (Benin, 2013), 9.6% (Burkina Faso 2013), 9.4% (Cote d’Ivoire, 2013), 8% (Guinea 2013), 13.5% (Mali 2013), 11.8% (Niger 2013) and 3.5% (Senegal 2013).

Since infant diarrhoea can be caused by the early introduction of formula and/or solid, semi-solid or soft foods, addressing the problem of early introduction of formula and/or solid, semi-solid or soft foods in these countries could go a long way to prevent or reduce the burden of morbidity and mortality, thereby promoting the achievement of the Millennium Development Goal (MGD) 4 of survival of the child. One way of addressing the problem of early introduction of solid, semi-solid or soft foods is by identifying factors associated with the practice.

This paper aimed to examine the prevalence of early introduction of formula and/or solid, semi-solid or soft foods to infants aged three to five months, and to identify the individual-, household- and community-level factors associated with this practice among such infants in seven Francophone West African countries. It is hoped that the results from our findings will be of assistance to governments and other stakeholders in the implementation of interventions to discourage early introduction of complementary feeding and thereby improve exclusive breastfeeding practices.

## 2. Experimental Section

Analyses conducted in this study were based on the most recent DHS data for the 7 countries (Benin, Burkina Faso, Cote d’Ivoire, Guinea, Mali, Niger and Senegal), collected between 2010 and 2013 [14,15,16,17,18,19,20,21]. These reports were downloaded from the public domain. The various DHS reports are nationally representative household surveys, which adopt a multistage stratified cluster sampling design. Analyses in this study were limited to infants aged between 3 and 5 months and living with the respondent (ever-married women aged 15–49 years). The total weighted sample sizes for the various countries were: 671 (Benin), 811 (Burkina Faso), 362 (Cote d’Ivoire), 398 (Guinea), 519 (Mali), 767 (Niger) and 630 (Senegal). Details of survey methodology, sampling procedure and questionnaire can be found in the respective DHS reports [14,15,16,17,18,19,20,21].

### 2.1. Outcome Indicator and Explanatory Variables

The outcome variable for this study (early introduction of formula and/or solid, semi-solid or soft foods) was defined as the proportion of infants aged between 3 and 5 months who received solid, semi-solid or soft foods during the past 24 h. This indicator was examined against a set of explanatory (independent) variables classified into 3 levels: individual, household and community. The individual-level attributes of the infant included their sex, age in completed months and size at birth as perceived by the mother. The individual-level characteristics of the mother included her age in years, work status, highest level of education achieved, religion, marital status and access to the media. Other individual-level characteristics involved the mother-infant dyad: number of antenatal clinic visits, place of delivery, mode of delivery, birth order, and timing of postnatal contacts with health-care providers.

Household-level characteristics comprised wealth index of the household and the source of drinking water. The household wealth index was constructed using a method recommended by the World Bank Poverty Network and United Nations Children’s Fund [22]. The index was divided into five quintiles, namely: *poorest*, *poorer*, *middle*, *richer* and *richest*. In this study, the index was re-categorized into *poor*, *middle* and *rich* to make the analysis easier.

The community-level characteristics comprised the type of residence (urban or rural) and the administrative or geographical region.

### 2.2. Statistical Analyses

Analyses were performed using Stata version 12.0 (Stata-Corp., College Station, TX, USA) “Svy” (survey) commands to allow for adjustments for the cluster sampling design. The calculation of standard errors using the Taylor series linearization method was used in the surveys when determining confidence intervals around prevalence estimates. Early introduction of formula and/or solid, semi-solid or soft foods was expressed as a dichotomous variable, and the significance of associations was tested with a Chi-squared (χ^2^) test.

Simple logistic regression methods were used to select individual, household and community variables with *p* < 0.20. Multiple logistic regression using backward stepwise (manually executed) method was used to eliminate the non-significant factors and determine the factors significantly associated with early introduction of solid, semi-solid or soft foods. In order to assess the adjusted risk of independent variables, the odds ratios with 95% confidence intervals were calculated. The variables with a *p*-value less than 0.05 were retained in the final model. Separate logistic regression models were run for each country, because the pattern of prevalence of early introduction of formula and/or solid, semi-solid or soft foods by the same variables varied across countries.

## 3. Results

### 3.1. Characteristics of the Samples

Table 1 summarizes the distribution of the individual-, household- and community-level characteristics of infants aged three to five months across the seven Francophone West African countries. In all seven countries, the majority of mothers were not engaged in paid employment. A very small proportion of mothers across all the countries had secondary or higher education. This ranged from a highest value of only 14.6% (Benin) to a very low value of 3.0% (Niger). The proportions of mothers with secondary education or higher for the rest of the countries were 13.3% (Cote d’Ivoire), 11.0% (Guinea), 10.7% (Mali), 8.8% (Senegal), and 5.6% (Burkina Faso). A large majority of mothers across all seven countries were currently married. The proportions ranged between 98.6% (Niger) and 84.0% (Cote d’Ivoire), with the rest of the values being 97.0% (Mali), 96.8% (Burkina Faso), 96.1% (Senegal), 94.5% (Benin) and 94.3% (Cote d’Ivoire). A high proportion of the infants across all the countries did not suffer any of the three illnesses (fever, diarrhoea and acute respiratory infection) in the 24 h prior to the surveys. In all the countries, the majority of mothers resided in rural areas. More than 90% of mothers had made at least one antenatal clinic visit during their pregnancy in Benin, Burkina Faso, Cote d’Ivoire and Senegal; and more than 70% of mothers had attended such clinics in the other countries. In all seven countries, more than 90% of births were through non-caesarean section.

**Table 1:** Individual-, household- and community-level characteristics of infants aged three to five months in seven Francophone West African countries, 2010–2013 (in percentages).

Characteristic	Benin	Burkina Faso	Cote d’Ivoire	Guinea	Mali	Niger	Senegal
	(*n* = 671)	(*n* = 811)	(*n* = 362)	(*n* = 398)	(*n* = 519)	(*n* = 767)	(*n* = 630)
*Individual-level factors*							
Mother’s work status							
Non-working	61.3	80.8	52.3	71.5	63.5	84.2	73.8
Working (past 12 months)	38.7	19.2	47.7	28.5	36.5	15.8	26.2
Father’s occupation							
Non Agricultural	52.8	28.5	37.7	33.7		46.8	73.4
Agricultural	41.9	71.5	44.1		66.3	53.2	26.6
Not working	5.3		18.2				
Mother’s education							
No education	67.4	83.5	65.0	76.2	82.2	87.4	74.6
Primary	17.9	10.9	21.7	12.8	7.1	9.6	16.6
Secondary and higher	14.6	5.6	13.3	11.0	10.7	3.0	8.8
Father’s education							
No education	56.2	82.0	50.8	68.6	80.5	78.6	80.2
Primary	19.4	11.0	23.8	8.5	7.0	13.2	10.8
Secondary and higher	24.4	7.0	25.4	22.9	12.5	8.2	9.0
Perceived size of baby at birth							
Small	12.8	14.1	15.4	10.7	16.5	28.0	34.1
Average	70.4	55.1	36.1	40.9	39.2	53.6	42.4
Large	16.8	30.8	48.5	48.4	44.3	18.4	23.5
Mother’s age (years)							
15–24	32.6	38.3	40.9	39.2	31.9	38.0	38.3
25–34	53.3	43.9	43.0	41.4	51.9	44.4	46.1
35–49	14.1	17.8	16.1	19.4	16.2	17.6	15.6
Marital status							
Currently married	94.5	96.8	84.0	94.3	97.0	98.6	96.1
Formerly married *	5.5	3.2	16.0	5.7	3.0	1.4	3.9
Mother’s religion							
Christian	55.5	35.1	45.6	11.4			2.7
Muslim and others	44.5	64.9	54.4	88.6			97.3
Birth order of infant							
First-born	22.1	17.7	20.2	18.4	14.8	14.4	22.3
2nd–4th	52.8	45.9	51.4	48.4	46.4	40.0	46.7
5 or more	25.1	36.4	28.4	33.2	38.8	45.6	31.0
Preceding birth interval							
No previous birth	22.1	17.7	20.2	18.4	14.8	14.4	22.4
<24 months	7.6	6.2	6.5	8.6	5.8	14.1	11.4
>24 months	70.3	76.1	73.3	73.0	79.4	71.5	66.2
Sex of baby							
Male	53.5	49.8	48.5	50.5	46.5	51.1	48.8
Female	46.5	50.2	51.5	49.5	53.5	48.9	51.2
Mode of delivery							
Non-caesarean	93.7	97.3	97.0	97.1	97.5	98.6	94.2
Caesarean	6.3	2.7	3.0	2.9	2.5	1.4	5.8
Mother’s BMI (kg/m^2^)							
≤18	4.0	6.2	1.8	4.9	4.2	6.9	14.7
18–25	71.4	84.9	73.5	77.6	80.9	77.9	71.8
>25	24.6	8.9	24.7	17.5	14.9	15.2	13.5
Mother’s age at child’s birth							
<20	10.2	11.9	14.7	20.0	14.5	14.7	15.6
20–29	54.4	52.5	56.1	46.1	48.3	53.2	49.4
30–39	31.8	30.1	25.4	29.8	33.0	27.1	32.1
>40	3.6	5.5	4.0	4.1	4.2	5.0	2.9
Place of delivery							
Home	11.4	25.5	41.4	61.2	41.2	66.5	28.9
Health facility	88.6	74.5	58.6	38.8	58.8	33.4	71.1
Infant had diarrhoea (in past 2 weeks)							
No	92.3	86.5	87.0	88.8	91.2	79.9	73.9
Yes	7.7	13.5	13.0	11.2	8.8	20.1	26.1
Infant had acute respiratory infection (in past 2 weeks)							
No	96.8	95.5	86.8	80.8	91.6	87.6	86.2
Yes	3.2	4.5	13.2	19.2	8.4	12.4	13.8
Infant had fever (in the past 2 weeks)							
No	90.0	81.0	78.5	72.4	95.9	82.6	74.9
Yes	10.0	19.0	21.5	27.6	4.1	17.4	25.1
Type of delivery assistance							
Health professional	80.0	71.5	59.6	42.2	60.7	32.6	53.8
Traditional birth attendant	4.0	6.6	19.0	33.9	22.7	33.6	10.3
Other untrained personnel	16.0	21.9	21.4	23.9	16.6	33.8	35.9
Antenatal Clinic visits							
None	9.9	4.3	7.1	11.3	23.8	11.8	4.5
1–3	28.0	63.2	54.1	30.0	38.6	55.4	46.2
4+	62.1	32.5	38.8	58.7	37.6	32.8	49.3
Timing of postnatal check-up							
No check-ups (including missing)	62.0	45.9	37.0	53.7	64.2	59.2	24.2
0–2 days	28.6	39.8	51.9	30.9	30.2	23.9	45.5
3–6 days	8.2	12.5	9.3	6.0	5.3	6.1	10.4
7+ days	1.2	1.8	1.8	9.4	0.3	10.8	19.9
Maternal literacy							
No	73.1	85.9	74.8	86.0	84.2	92.0	78.3
Yes	26.9	14.1	25.2	14.0	15.8	8.0	21.7
Mother’s access to newspaper/magazine							
No	93.6	96.9	88.3	95.8	94.5	98.0	89.6
Yes	6.4	3.1	11.7	4.2	5.5	2.0	10.4
Mother’s access to the radio							
No	36.3	29.9	56.6	38.5	22.4	35.0	21.7
Yes	63.7	70.1	43.4	61.5	77.6	65.0	78.3
Mother’s access to television							
No	55.9	77.1	41.4	61.8	52.1	79.5	41.2
Yes	44.1	22.9	58.6	38.2	47.9	20.5	58.8
*Household-level factors*							
Household Wealth Index							
Poor	40.5	44.4	43.7	47.1	41.8	40.4	53.6
Middle	39.1	43.5	41.6	39.3	40.6	43.5	33.4
Rich	20.4	12.1	14.7	13.7	17.6	16.1	13.0
Source of drinking water							
Unprotected	23.5	23.8	25.1	34.4	37.6	37.8	28.7
Protected	76.5	76.2	74.9	65.6	62.4	62.2	71.3
*Community-level factors*							
Residence							
Urban	39.7	15.2	38.6	25.3	19.7	13.3	32.4
Rural	60.3	84.8	61.4	74.7	80.3	86.7	67.6
Geographical/Administrative region							
1	6.2	10.2	6.6	7.5	14.4	1.0	12.6
2	10.6	4.8	2.4	12.7	21.2	2.0	4.3
3	10.5	8.1	7.9	11.1	21.0	11.1	15.5
4	7.1	8.1	14.8	20.6	19.4	22.7	6.8
5	7.2	9.1	4.3	10.9	13.8	22.6	8.1
6	7.8	9.3	4.7	10.2	10.2	10.8	11.8
7	4.1	3.2	5.9	7.3		24.2	14.6
8	12.8	12.0	12.6	19.7		5.6	6.0
9	4.1	10.1	14.3				8.0
10	12.6	7.9	11.6				7.1
11	7.9	3.6	14.9				5.2
12	9.1	9.6					
13		4.1					

Geographical/Administrative region 1 = Alibori (Benin), Boucle de Mouhoun (Burkina Faso), Centre (Cote d’Ivoire), Boké (Guinea), Agadez (Niger), Kayes (Mali) and Daka (Senegal); 2 = Atacora (Benin), Cascades (Burkina Faso), Centre-Est (Cote d’Ivoire), Conakry (Guinea), Diffa (Niger), Koulikor (Mali) and Diourbel (Senegal); 3 = Atlanti (Benin), Centre (Burkina Faso), Centre-Nord (Cote d’Ivoire), Faranah (Guinea), Dosso (Niger), Sikasso (Mali) and Fatick (Senegal); 4 = Borgou (Benin), Centre-Est (Burkina Faso), Centre-Ouest (Cote d’Ivoire), Kankan (Guinea), Maradi (Niger), Segou (Mali) and Kaolack (Senegal); 5 = Colline (Benin), Centre-nord (Burkina Faso), Nord (Cote d’Ivoire), Kindia (Guinea), Tahoua (Niger), Mopti (Mali) and Kolda (Senegal); 6 = Couffo (Benin), Centre-ouest (Burkina Faso), Nord-est (Cote d’Ivoire), Labé (Guinea), Tillabér (Niger), Bamako (Mali) and Louga (Senegal); 7 = Donga (Benin), Centre-sud (Burkina Faso), Nord-Ouest (Cote d’Ivoire), Mamou (Guinea), Zinder (Niger) and Matam (Senegal); 8 = Littoral (Benin), Est (Burkina Faso), Ouest (Cote d’Ivoire), N’zéréko (Guinea), Niamey (Niger) and Saint-Louis (Senegal); 9 = Mono (Benin), Hauts Bassins (Burkina Faso), Sud sans Abidjan (Cote d’Ivoire) and Tambacounda (Senegal); 10 = Quémé (Benin), Nord (Burkina Faso), Sud-ouest (Cote d’Ivoire) and Thiès (Senegal); 11 = Plateau (Benin), Plateau Central (Burkina Faso) Ville d’Abidjan (Cote d’Ivoire) and Zuguinchor (Senegal); 12 = Zou (Benin), Sahel (Burkina Faso); 13 = Sud-Ouest (Burkina Faso); * Included divorced, separated or widowed.

### 3.2. Early Introduction of Formula and/or Solid, Semi-Solid or Soft Foods

As shown in Table 2, the percentage of early introduction of formula and/or solid, semi-solid or soft foods (EISF) was not significantly different between working and non-working mothers across all seven countries. The percentage of EISF among infants from Burkina Faso and Guinea whose mothers had secondary education or higher was greater than those whose mothers had lower levels of education. In the rest of the countries, infants whose mothers had secondary education or higher had relatively lower percentages of EISF compared to those whose mothers had lower levels of education, although the differences were not statistically significant. Infants whose fathers had secondary or higher level of education showed higher percentages of EISF in Burkina Faso, Guinea and Mali compared to their counterparts whose fathers had lower levels of education. The percentage of EISF was lower among infants whose fathers had secondary or higher level of education in the other countries, although these differences were not significant. However, in Benin, infants whose fathers had secondary or higher level of education had significantly lower percentage of EISF. In all seven countries, second- or higher-born infants showed higher percentages of EISF, although the differences were not significant. With the exception of Benin, male infants in all the other countries showed higher EISF percentages compared to their female counterparts. These differences were statistically significant in Cote d’Ivoire and Senegal. However, the differences were not significant in the rest of the countries. Mothers in Benin, Cote d’Ivoire and Guinea whose infants contracted diarrhoea during the past two weeks reported significantly higher percentages of EISF, compared to those whose infants did not have this illness. Mothers in Benin, Guinea and Senegal whose infants had acute respiratory infection during the past two weeks reported significantly higher percentages of EISF compared to those whose infants did not have the illness. In Benin, Guinea and Senegal, mothers whose infants developed fever during the past two weeks reported higher percentages of EISF. The differences, however, were significant only in Benin and Guinea. The results were different in Mali and Niger, where mothers whose infants did not have fever during the past two weeks reported higher percentages of EISF. Mothers in Benin, Burkina Faso, Niger and Senegal, whose babies were delivered with the help of traditional birth attendants reported higher percentages of EISF than those from the other countries. The differences, however, were significant only in Mali, Niger and Senegal. The prevalence of EISF was significantly higher among infants in Burkina Faso and Niger, whose mothers did not attend any antenatal clinics, compared to those whose mothers had at least one antenatal clinic visit. In the rest of the countries, mothers who had at least one antenatal clinic visit reported higher prevalence of EISF, compared to those who did not attend any antenatal clinic. The prevalence of EISF was higher among infants in Burkina Faso, Cote d’Ivoire, Guinea, Mali and Senegal, whose mothers resided in urban areas, compared to those who resided in rural areas. The results, however, were significant for only Cote d’Ivoire and Senegal. Mothers in the rest of the countries who resided in rural areas reported high prevalence of EISF compared to their urban counterparts; although these differences were not significant.

**Table 2:** Early introduction of solid, semi-solid or soft foods among infants aged three to five months by individual-, household- and community-level characteristics in seven Francophone West African countries, 2010–2013.

Characteristic	Benin	Burkina Faso	Cote d’Ivoire	Guinea	Mali	Niger	Senegal
	%[95% CI]	%[95% CI]	%[95% CI]	%[95% CI]	%[95% CI]	%[95% CI]	%[95% CI]
*Individual-level factors*							
Mother’s working status							
Non-working	32.7[26.7,39.2]	5.6[2.6,11.8]	19.3[13.1,27.6]	15.3[8.5,25.9]	23.6[18.6,29.4]	11.6[8.8,15.2]	18.9[15.2,23.3]
Working (past 12 months)	27.9[23.6,32.6]	7.4[5.3,10.2]	25.0[19.2,32.0]	10.7[7.4,15.1]	19.1[13.4,26.5]	18.7[12.8,26.7]	23.0[15.6,32.6]
Mother’s education							
No education	31.1[26.7,35.8]	7.2[5.1,10.1]	23.1[17.2,30.4]	11.6[8.3,16.1]	22.6[18.2,27.7]	13.5[10.7,17.0]	17.8[14.3,21.9]
Primary	31.7[23.5,41.1]	3.2[0.8,11.8]	20.8[11.9,33.8]	6.7[2.2,18.7]	20.4[9.5,38.5]	7.7[3.3,17.0]	29.5[18.0,44.3]
Secondary and higher	21.0[13.7,30.9]	11.9[4.6,27.5]	20.8[9.5,39.6]	20.6[7.2,46.4]	18.0[9.1,32.5]	6.8[2.4,17.9]	21.2[10.6,38.0]
Father’s education							
No education	30.6[25.8,35.9] ^§^	6.7[4.8,9.5]	23.1[15.4,33.2]	10.6[7.3,15.2]	23.0[18.4,28.4]	13.2[10.2,17.0]	19.4[15.6,23.9]
Primary	38.6[30.1,47.8]	6.3[2.4,15.5]	27.6[17.7,40.5]	12.9[5.6,27.0]	19.4[10.0,34.4]	11.9[6.6,20.5]	30.4[16.9,48.4]
Secondary and higher	26.0[18.5,35.3]	10.8[4.6,23.4]	22.7[13.4,35.7]	17.0[8.6,30.8]	17.9[9.6,30.9]	11.5[5.5,22.4]	16.7[8.6,29.9]
Size of baby at birth							
Small	24.9[17.0,34.8]	6.1[2.4,14.6]	12.8[5.8,26.0]	10.1[3.8,24.5]	17.8[10.9,27.7]	12.5[8.2,18.6]	20.5[14.7,27.8]
Average	31.6[27.1,36.5]	7.3[4.9,10.6]	28.4[20.0,38.6]	13.5[9.1,19.4]	25.0[18.5,32.8]	13.3[9.9,17.8]	19.6[14.7,25.6]
Large	26.0[18.2,35.8]	7.1[4.2,11.8]	20.9[14.8,28.6]	11.1[6.4,18.7]	21.1[15.7,27.9]	12.1[7.2,19.8]	20.6[14.1,29.2]
Mother’s age (years)							
15–24	26.0[20.7,32.2]	7.6[4.7,12.2]	23.6[16.5,32.5]	10.3[5.3,19.1]	14.9[9.9,21.9]	10.6[7.2,15.3] ^§^	22.5[16.5,29.9]
25–34	32.3[27.3,37.8]	6.1[3.9,9.6]	19.8[13.6,27.9]	11.8[7.6,17.9]	27.4[21.3,34.6]	13.8[9.7,19.3]	17.6[13.4,22.9]
35–49	28.4[19.6,39.1]	8.0[3.5,17.1]	25.9[14.3,42.3]	15.7[8.8,26.5]	18.2[11.0,28.5]	14.6[9.3,22.3]	21.0[13.2,31.6]
Marital status							
Currently married	30.2[26.6,34.1]	7.0[5.1,9.6]	22.9[18.0,28.8]	12.5[9.0,17.1]	21.9[18.0,26.5]	12.9[10.3,16.1]	19.5[16.0,23.4]
Formerly married *	21.5[11.0,37.9]	8.2[1.2,40.1]	19.1[9.7,34.2]	3.1[0.4,19.4]	22.2[6.4,54.1]	0	33.9[18.2,54.1]
Mother’s religion							
Christian	30.0[24.9,35.5]	7.7[4.9,11.9]	19.4[13.4,27.3]	5.7[1.5,19.5]	10.2[3.14,28.39]		24.3[10.1,47.6]
Muslim and others	29.4[24.4,35.0]	6.7[4.3,10.3]	24.7[18.0,32.9]	12.7[9.2,17.3]	8.7[6.57,11.43]		19.9[16.4,23.9]
Birth order							
First-born	23.0[16.8,30.5]	11.6[6.1,21.1]	19.2[11.1,31.2]	7.4[3.4,15.7]	11.7[6.3,20.8]	8.4[4.2,16.0]	22.0[14.2,32.4]
2nd-4th	32.2[27.1,37.9]	5.4[3.5,8.4]	25.3[19.2,32.6]	11.3[6.5,18.8]	24.4[18.7,31.2]	13.3[9.5,18.2]	19.3[14.6,25.1]
5 or more	30.4[23.5,38.3]	6.9[4.0,11.6]	19.0[11.5,29.8]	15.5[9.8,23.6]	22.9[17.3,29.7]	13.7[9.8,18.7]	19.7[14.6,26.1]
Preceding birth interval							
No previous birth	23.0[16.8,30.5]	11.6[6.1,21.1]	19.2[11.1,31.2]	7.4[3.4,15.7]	11.7[6.3,20.8]	8.4[4.2,16.0]	22.0[14.2,32.4]
<24 months	25.2[13.8,41.6]	4.0[1.0,15.3]	24.6[9.3,51.0]	11.6[4.0,29.0]	22.6[9.4,44.8]	13.1[7.5,21.8]	21.5[13.0,33.3]
>24 months	32.4[28.1,37.1]	6.2[4.4,8.8]	23.0[17.9,28.9]	13.2[9.3,18.5]	23.8[19.2,29.2]	13.6[10.3,17.7]	19.0[15.2,23.3]
Sex of baby							
Male	32.3[27.5,37.4]	8.2[5.6,11.7]	28.6[21.2,37.4] ^§^	14.1[9.5,20.4]	23.3[18.2,29.3]	14.8[10.6,20.2]	25.2[19.5,31.9] ^§^
Female	26.8[21.8,32.5]	5.9[3.8,9.2]	16.4[11.4,23.0]	9.8[6.2,15.0]	20.7[15.8,26.8]	10.6[7.7,14.5]	15.1[11.3,19.8]
Mode of delivery							
Non-caesarean	30.0[26.3,33.9]	7.2[5.3,9.9]	21.7[17.2,27.0]	11.9[8.5,16.3]	21.8[17.8,26.4]	12.9[10.3,16.1]	20.2[16.7,24.3]
Caesarean	26.1[13.8,44.0]		42.3[13.6,77.4]	15.5[3.4,49.4]	26.9[7.0,64.4]	0	16.4[4.5,45.2]
Mother’s BMI (kg/m^2^)							
≤18	24.3[11.8,43.6]	1.7[0.2,11.5]	72.4[24.1,95.6]	21.1[3.2,68.6]	24.7[7.7,56.3]	12.7[2.8,42.5]	10.0[3.4,25.9]
18–25	30.2[26.0,34.7]	5.1[3.0,8.6]	18.7[12.2,27.5]	12.5[8.4,18.3]	22.1[15.7,30.2]	10.5[7.0,15.5]	22.1[15.6,30.3]
>25	29.0[21.6,37.8]	7.2[2.0,23.1]	24.0[12.2,41.8]	12.4[3.9,33.0]	20.3[10.0,37.0]	18.5[10.3,31.0]	29.5[14.8,50.3]
Mother’s age at child’s birth							
<20	25.1[16.7,35.9]	7.0[2.9,16.0]	23.4[12.3,39.9]	10.0[4.5,20.5]	15.5[8.3,27.3]	6.3[2.9,13.2]	18.2[10.4,29.7]
20–29	28.3[23.5,33.7]	6.7[4.3,10.3]	22.2[16.6,29.0]	11.8[7.5,18.2]	19.8[14.7,26.2]	12.8[9.3,17.3]	19.6[15.1,25.0]
30–39	32.3[25.8,39.7]	6.7[3.9,11.2]	21.2[13.0,32.7]	14.6[8.7,23.4]	28.8[21.9,36.8]	16.0[11.0,22.7]	20.7[13.7,30.1]
>40	41.2[24.9,59.6]	12.2[4.3,30.1]	26.9[8.2,60.2]	4.5[0.6,27.6]	15.0[4.6,39.2]	13.9[5.5,31.1]	29.6[13.3,53.7]
Place of delivery							
Home	33.7[24.5,44.3]	6.9[4.1,11.4]	19.5[12.6,28.9]	12.4[8.8,17.3]	27.0[20.3,34.9]	13.8[10.5,18.0]	18.9[13.9,25.1]
Health facility	29.2[25.4,33.4]	7.1[4.8,10.3]	24.3[18.8,30.8]	11.3[6.1,19.9]	18.4[13.7,24.3]	10.6[7.3,15.3]	20.5[16.2,25.6]
Infant had diarrhea (in past 2 weeks)							
No	28.3[24.7,32.2] ^§^	7.3[5.2,10.0]	20.1[15.4,25.8] ^§^	10.7[7.7,14.6] ^§^	21.9[17.8,26.8]	11.9[9.3,15.2]	19.5[15.9,23.7]
Yes	47.0[33.0,61.6]	5.7[2.7,11.4]	37.3[22.7,54.7]	22.0[11.2,38.9]	22.2[11.4,38.9]	16.0[10.6,23.5]	21.5[14.9,30.0]
Infant had acute respiratory infection (in past 2 weeks)							
No	28.7[25.1,32.5] ^§^	7.2[5.2,9.9]	21.3[16.6,27.0]	8.9[6.1,12.8] ^§^	21.9[17.8,26.6]	12.6[9.7,16.1]	18.0[14.8,21.6] ^§^
Yes	61.5[36.9,81.4]	3.3[0.8,12.2]	28.7[15.5,46.8]	24.8[13.9,40.2]	23.6[10.7,44.4]	14.0[7.3,25.0]	32.6[20.9,47.1]
Infant had fever (in the past 2 weeks)							
No	28.0[24.4,31.9] ^§^	6.5[4.7,9.1]	20.0[15.2,25.8]	9.2[6.3,13.3] ^§ ^	22.2[18.2,26.8]	13.2[10.2,16.8]	19.2[15.6,23.4]
Yes	45.2 [32.3,58.7]	9.2[5.2,15.9]	30.9[20.1,44.3]	19.2[11.4,30.3]	16.0[4.6,43.0]	10.7[5.4,20.0]	22.5[15.3,31.7]
Type of delivery assistance							
Health professional	30.1[26.1,34.4]	6.7[4.5,9.8]	25.3[19.8,31.8]	11.4[6.1,20.3]	19.3[14.3,25.6] ^§^	9.4[6.3,13.7] ^§^	21.4[16.4,27.4] ^§^
Traditional birth attendant	42.4[26.3,60.3]	9.5[3.8,22.1]	21.5[12.1,35.3]	9.0[4.8,16.4]	34.6[25.0,45.7]	18.1[12.6,25.4]	32.7[22.0,45.5]
Other untrained personnel	25.0[17.2,34.9]	7.6[4.2,13.4]	16.6[9.0,28.5]	15.3[9.2,24.3]	14.2[7.8,24.4]	10.6[7.1,15.5]	15.8[10.8,22.5]
Antenatal Clinic visits							
None	28.9[19.6,40.4]	24.4[11.8,43.8] ^§^	19.8[6.3,47.6]	15.9[7.5,30.6]	28.5[20.1,38.8]	26.9[16.3,40.9] ^§^	20.1[10.6,34.7]
1–3	31.6[25.2,38.8]	4.8[3.0,7.4]	20.6[15.2,27.4]	9.8[5.3,17.6]	18.7[12.8,26.3]	11.8[8.6,15.9]	19.0[14.9,23.8]
4+	30.0[25.3,35.3]	9.2[5.7,14.4]	25.4[17.7,34.9]	12.4[8.0,18.9]	21.3[15.7,28.2]	9.4[6.1,14.2]	20.4[14.9,27.3]
Timing of postnatal check-up							
No check-ups ‡	31.5[24.5,39.4]	7.8[5.2,11.6]	22.3[14.6,32.6]	11.0[7.3,16.4]	24.3[19.2,30.2]	12.6[9.1,17.2]	24.6[18.4,32.1]
0–2 days	34.5[22.2,49.5]	6.7[4.0,11.1]	22.9[16.9,30.3]	14.0[7.5,24.4]	18.0[12.1,25.9]	13.6[9.3,19.5]	19.7[14.6,26.0]
3–6 days	33.6[12.8,63.5]	6.4[2.7,14.3]	17.2[6.8,37.2]	5.9[0.8,32.7]	17.7[6.8,38.8]	12.6[5.0,28.4]	10.9[3.7,28.0]
7+ days	28.2[23.8,33.1]		32.9[5.1,81.8]	14.9[6.4,31.0]		11.6[6.2,20.6]	20.0[12.7,30.0]
Maternal literacy							
No	30.7[26.6,35.1]	7.4[5.3,10.4]	23.2[17.7,29.8]	11.0[8.0,15.1]	22.7[18.4,27.7]	13.2[10.4,16.5]	19.6[15.9,24.0]
Yes	27.1[20.4,35.0]	4.7[1.8,12.0]	19.6[11.4,31.6]	17.9[6.9,39.2]	17.8[10.4,28.9]	7.7[3.2,17.4]	21.5[14.4,30.8]
Mother’s access to newspaper/magazine							
No	30.1[26.3,34.1]	7.0[5.1,9.7]	22.6[17.7,28.4]	11.6[8.6,15.5]	22.1[18.0,26.9]	12.9[10.3,16.1]	19.5[15.8,23.8]
Yes	25.0[14.4,39.7]	7.4[2.2,22.6]	19.8[8.3,40.2]	20.3[5.3,53.8]	18.6[8.8,35.0]	4.6[1.1,17.7]	24.8[14.1,39.8]
Mother’s access to the radio							
No	33.4[27.7,39.6]	7.0[4.1,11.8]	23.3[17.2,30.6]	9.0[5.3,15.1]	27.2[19.6,36.2]	11.6[7.7,17.0]	16.7[9.7,27.1]
Yes	[23.3,32.4]	7.1[4.9,10.1]	21.1[14.7,29.2]	13.8[9.7,19.3]	19.5[15.3,24.5]	13.4[10.1,17.5]	20.9[17.2,25.3]
Mother’s access to television							
No	29.0[24.5,34.0]	6.8[4.7,9.8]	16.3[10.7,24.1]	10.7[7.2,15.7]	25.0[19.1,32.0]	11.4[8.7,14.9]	17.7[13.9,22.4]
Yes	30.6[25.0,36.8]	7.7[4.0,14.5]	26.6[20.3,33.9]	14.0[8.4,22.5]	18.6[13.9,24.5]	17.9[11.0,27.7]	21.6[16.6,27.6]
*Household-level factors*							
Household Wealth Index							
Poor	28.1[22.9,34.0]	5.8[3.7,8.9]	12.0[7.9,17.9] ^§^	11.1[7.1,16.8]	25.7[19.0,33.8]	14.9[10.4,21.0]	17.9[14.6,21.8]
Middle	32.5[27.2,38.2]	8.6[5.5,13.2]	29.9[21.6,39.7]	13.2[7.2,23.2]	17.7[12.6,24.4]	9.6[5.6,15.8]	20.4[13.5,29.6]
Rich	27.7[19.0,38.4]	6.1[2.2,15.6]	31.6[20.7,44.8]	12.5[6.5,22.7]	22.7[15.3,32.3]	12.2[8.7,16.7]	23.5[15.9,33.4]
*Community-level factors*							
Residence							
Urban	29.1[23.2,35.9]	9.3[4.2,19.2]	29.2[22.1,37.4] ^§^	13.3 [6.4,25.9]	23.0[15.6,32.4]	10.8[6.5,17.5]	27.9[20.11,37.22] ^§^
Rural	30.1 [25.7,34.9]	6.6[4.7,9.3]	18.0[12.6,25.1]	11.5 [8.2,15.9]	21.7[17.2,27.0]	13.0[10.2,16.6]	16.3[13.1,20.0]
Geographical/Administrative region							
1	17.7[8.9,32.2]	0	15.8[5.8,36.6]		17.4[11.0,26.4]	4.2[0.5,26.6]	34.2[18.3,54.7] ^§^
2	29.5[20.4,40.7]	6.6[2.9,14.5] ^§^	11.1[2.3,40.1]	15.4[4.9,39.0] ^§^	14.4[7.9,24.8]	34.1[18.0,55.0]	44.3[27.2,62.8]
3	29.5[19.2,42.4]	18.4[8.9,34.2]	14.9[6.0,32.8]	8.0[3.6,16.6]	29.8[19.8,42.1]	14.7[8.7,23.7]	16.3[9.3,27.0]
4	23.8[12.8,39.8]	15.2[6.1,33.1]	27.5[15.5,43.8]	6.7[2.7,15.9]	18.5[10.0,31.7]	8.9[5.2,14.9]	11.3[4.2,27.0]
5	40.5[28.0,54.4]	10.2[4.8,20.4]	18.9[6.9,42.3]	3.9[0.5,24.8]	28.9[19.5,40.5]	8.6[3.9,18.0]	22.6[14.6,33.2]
6	46.3[33.4,59.7]	11.2[4.0,27.8]	13.7[5.2,31.8]	44.2[28.3,61.4]	25.2[16.3,36.8]	13.2[7.2,23.2]	15.6[8.9,25.9]
7	17.1[8.83,30.6]	11.4[4.3,27.3]	23.0[15.0,33.7]	10.1[3.4,26.6]	10.7[2.80,33.48]	17.6[11.4,26.1]	13.3[5.7,27.9]
8	32.9[20.2,48.7]	3.0[0.9,9.4]	21.8[9.2,43.5]	10.7[5.5,19.8]	59.3[31.09,82.46]	13.5[7.0,24.4]	7.0[1.6,26.4]
9	26.4[12.4,47.7]	1.8[0.2,11.8]	19.6[9.1,37.5]		16.2[9.85,25.36]		22.7[13.8,35.0]
10	21.2[13.5,31.6]	0	11.4[4.5,25.8]				26.9[16.9,39.9]
11	35.2[21.2,52.2]	7.1[2.3,19.5]	40.8[28.2,54.7]				15.4[7.2,29.9]
12	30.2[20.6,41.7]	2.9[0.7,10.9]					
13		15.2[7.5,28.2]					

Geographical/Administrative region 1 = Alibori (Benin), Boucle de Mouhoun (Burkina Faso), Boké (Guinea), Agadez (Niger), Kayes (Mali) and Daka (Senegal); 2 = Atacora (Benin), Cascades (Burkina Faso), Conakry (Guinea), Diffa (Niger), Koulikor (Mali) and Diourbel (Senegal); 3 = Atlanti (Benin), Centre (Burkina Faso), Faranah (Guinea), Dosso (Niger), Sikasso (Mali) and Fatick (Senegal); 4 = Borgou (Benin), Centre-Est (Burkina Faso), Kankan (Guinea), Maradi (Niger), Segou (Mali) and Kaolack (Senegal); 5 = Colline (Benin), Centre-nord (Burkina Faso), Kindia (Guinea), Tahoua (Niger), Mopti (Mali) and Kolda (Senegal); 6 = Couffo (Benin), Centre-ouest (Burkina Faso), Labé (Guinea), Tillabér (Niger), Tombouct (Mali) and Louga (Senegal); 7 = Donga (Benin), Centre-sud (Burkina Faso), Mamou (Guinea), Zinder (Niger), Gao (Mali) and Matam (Senegal); 8 = Littoral (Benin), Est (Burkina Faso), N’zéréko (Guinea), Niamey (Niger), Kidal (Mali) and Saint-Louis (Senegal); 9 = Mono (Benin), Hauts Bassins (Burkina Faso), Bamako (Mali) and Tambacounda (Senegal); 10 = Quémé (Benin), Nord (Burkina Faso) and Thiès (Senegal); 11 = Plateau (Benin), Plateau Central (Burkina Faso) and Zuguinchor (Senegal); 12 = Zou (Benin), Sahel (Burkina Faso); 13 = Sud-Ouest (Burkina Faso); * Included divorced, separated or widowed; § Statistically significant; ‡ Including missing values.

### 3.3. Factors Associated with Early Introduction of Formula and/or Solid, Semi-Solid or Soft Foods

The adjusted odds ratios for factors significantly associated with EISF based on multiple statistical analyses are presented in Table 3. Infants who contracted diarrhoea in the past two weeks had a higher likelihood of EISF in Benin. Those who contracted ARI in the past two weeks had a significantly higher association with EISF in Guinea and Senegal. The association of EISF was significantly higher among infants whose mothers did not have any antenatal clinic visits in Burkina Faso and Niger. Birth order of the infant was significantly associated with EISF, with second to fourth-born infants having a higher likelihood in Benin. Infants who were delivered with assistance from Traditional Birth Attendants (TBA) had a significantly higher association with EISF in Niger and Senegal. Other factors significantly associated with EISF in the individual countries were unemployed fathers (Benin), male infants (Cote d’Ivoire and Senegal), working mothers (Niger), wealthy households (Cote d’Ivoire) and infants born to urban mothers (Senegal).

**Table 3:** Factors associated with early introduction of solid, semi-solid or soft foods among infants aged three to five months in seven Francophone West African countries, 2010–2013.

Country	Variable	OR	(95% CI)	*p* value
Benin	Child had diarrhoea (in past 2 weeks)			
	No	1.00		
	Yes	1.95	[1.01,3.76]	0.046
	Administrative region	See details below ^a^		
	Father’s occupation			
	Non Agricultural	1.00		
	Agricultural	2.25	[0.90,5.60]	0.082
	Not working	2.65	[1.03,6.84]	0.043
	Birth order			
	First-born	1.00		
	2nd-4th	1.60	[1.00,2.55]	0.050
	5 or more	1.52	[0.86,2.67]	0.148
	Age of child (months)	1.56	[1.23,1.97]	<0.001
Burkina Faso	Antenatal Clinic visits			
	None	1.00		
	1–3	0.13	[0.05,0.36]	<0.001
	4+	0.25	[0.09,0.69]	0.008
	Age of child (months)	2.26	[1.47,3.49]	<0.001
Cote d’Ivoire	Household Wealth Index			
	Poor	1.00		
	Middle	3.61	[1.84,7.12]	<0.001
	Rich	4.7	[2.02,10.90]	<0.001
	Sex of baby			
	Male	1.00		
	Female	0.36	[0.19,0.69]	0.002
	Age of child (months)	1.86	[1.29,2.69]	0.001
Guinea	Acute respiratory infection (in past 2 weeks)			
	No	1.00		
	Yes	3.21	[1.34,7.65]	0.009
	Age of child (months)	1.72	[1.10,2.70]	0.017
Mali	Type of delivery assistance			
	Health professional	1.00		
	Traditional birth attendant	2.08	[1.14,3.80]	0.017
	Other untrained personnel	0.64	[0.30,1.37]	0.253
	Mother’s age (years)			
	15–24	1.00		
	25–34	2.11	[1.18,3.77]	0.012
	35–49	1.28	[0.63,2.59]	0.498
Niger	Type of delivery assistance			
	Health professional	1.00		
	Traditional birth attendant	2.03	[1.16,3.56]	0.014
	Other untrained personnel	1.08	[0.57,2.08]	0.808
	Mother’s work status			
	Non-working	1.00		
	Working (past 12 months)	2.12	[1.15,3.90]	0.016
	Antenatal Clinic visits			
	None	1.00		
	1–3	0.39	[0.19,0.82]	0.013
	4+	0.27	[0.12,0.58]	0.001
	Age of child (months)	2.20	[1.53,3.16]	<0.001
Senegal	Acute respiratory infection (in past 2 weeks)			
	No	1.00		
	Yes	2.2	[1.20,4.01]	0.011
	Sex of baby			
	Male	1.00		
	Female	0.56	[0.34,0.93]	0.025
	Type of delivery assistance			
	Health professional	1.00		
	Traditional birth attendant	2.71	[1.34,5.49]	0.006
	Other untrained personnel	0.81	[0.47,1.40]	0.446
	Age of child (months)	1.76	[1.30,2.38]	<0.001
	Residence			
	Urban	1.00		
	Rural	0.47	[0.27,0.82]	0.008

^a^ Compared to Alibori region, the odds of early introduction of complementary foods was significantly higher in Atacora [OR = 2.66; 95% CI: (0.96,7.36)], Atlanti [OR = 2.67; 95% CI: (0.90,7.92)], Borgou [OR = 2.08; 95% CI: (0.64,6.73)], Colline [OR = 4.06; 95% CI: (1.40,11.75)], Couffo [OR = 5.92; 95% CI: (2.07, 16.92)], Donga [OR = 1.32; 95% CI: (0.41,4.24)], Littoral [OR = 3.22; 95% CI: (1.03,10.06)], Mono [OR = 2.52; 95% CI: (0.68,9.32)], Quémé [OR = 1.76; 95% CI: (0.58,5.34)], Plateau [OR = 3.71; 95% CI: (1.21,11.40)] and Zou [OR = 2.76; 95% CI: (0.98,7.83)] regions in Benin.

## 4. Discussion

Mothers who do not practise exclusive breastfeeding for the first 6 months of an infant’s life are much more likely to have practised EISF. Factors associated with non-exclusive breastfeeding would be similar to those associated with EISF. In this study, factors that were significantly associated with EISF among infants aged three to five months across the seven Francophone West African countries included contraction of illnesses, maternal attendance of antenatal clinics, type of residence, mode of delivery and gender of the baby as well as birth order of the infant. The study found that there was a higher likelihood for EISF among infants who contracted diarrhoea and ARI in the two weeks preceding the survey. Non-attendance of antenatal clinic by mothers, mothers in paid employment, male infants and delivery of babies by TBAs were the other major factors that were significantly associated with EISF across these countries.

Past studies in some advanced countries have found significant differences in the timing of the introduction of solids according to different socio-demographic and lifestyle factors. These studies found that younger mothers [11,18,23,24,25,26] and mothers who smoked [18,25,27] were most likely to practise EISF. One of the findings in this study was that in Cote d’Ivoire and Niger, being a male infant was a significant predictor of EISF. This finding is consistent with a previous study in Scotland [24] in which the early introduction of solids was found to be associated with male babies. One explanation of this finding may be that male infants are likely to have a higher birth weight which is positively associated with early introduction of solids [28].

Previous studies have shown that early introduction of solids was associated with an increased incidence of respiratory illness at 14–26 weeks of age and persistent cough at 14–26 weeks and 27–39 weeks [28], and this was confirmed by our study, which found that there was a higher likelihood of EISF among infants who contracted ARI in the 24 h preceding the survey in Guinea and Senegal. There was a significant association between EISF and diarrhoea among children in Benin. This finding was consistent with a previous study [29] in Vietnam.

There is evidence in the extant literature of a high likelihood of nonexclusive breastfeeding among working mothers [30,31]. This evidence indicates that the likelihood of EISF among infants whose mothers were in paid employment was high. The finding was confirmed by our study, which found working mothers to be significantly more likely to practise EISF in Niger. This suggests the reinforcement of policies that promote optimum breastfeeding practices to include working mothers as a specific target. Interventions to support breastfeeding among working mothers could include an increased period of maternity leave to cover the recommended duration of exclusive breastfeeding [30], or provision of special facilities at work places for nursing mothers to breastfeed. This suggestion, however, may be quite impracticable, especially in developing countries.

EISF was found to be significantly associated with household wealth in Cote d’Ivoire. Infants from richer households were found to be significantly more likely to receive EISF than poorer households, consistent with a previous study [30], where better socio-economic status of households was found to be a negative factor for exclusive breastfeeding. Separate studies [7,32] also document lower rates of exclusive breastfeeding (higher rates of EISF) among women of higher income and social class. This scenario may arise because rich and well-educated families give breast milk substitutes, bottle-feeding, pre-lacteals and other early supplemental foods to infants.

As expected, increasing infant age was found to be consistently associated with significantly high EISF rates in all seven countries. A similar finding has been reported from a study across five East and Southeast Asian countries [30]. In that study, increasing infant age was found to be significantly associated with low exclusive breastfeeding rates, and therefore high EISF rates. The East and Southeast Asian countries [30] study revealed that a higher number of antenatal clinic visits made by the mother was significantly associated with non-exclusive breastfeeding in the Philippines, and the opposite was found to be true in Indonesia. In our study, we found that infants whose mothers made no antenatal clinic visits in Burkina Faso and Niger were significantly more likely to receive EISF. In Benin and Mali, we found that second to fourth-born infants (higher birth order infants) were significantly more likely to receive EISF compared to first-borns. This was in agreement with findings from a separate study [30] in which non-exclusive breastfeeding was found to be associated with higher birth order of the infant. A previous study in Malawi [33] found that delivery at a health facility was positively associated with exclusive breastfeeding (negatively associated with EISF), and evidence from another study in Sri Lanka [34] also supported the finding that antenatal contacts with health-care workers was related to improved breastfeeding practices. Our analysis revealed that infants who were delivered by TBAs were significantly more likely to receive EISF. Obviously, delivery by a TBA meant delivery outside of a health facility. There is therefore the need for health-care institutions and care providers (including TBAs) to be well educated about proper infant feeding practices so that they can in turn impart their knowledge to mothers who come in contact with them. It is suggested that all health care workers involved in care for mothers and babies should adhere to WHO/UNICEF recommended training courses for lactation management [30]. This could also be supplemented by health worker follow-up through home-based postnatal care and to ensure proper breastfeeding practices and to address any breastfeeding issues [35].

This study has a number of strengths. The data on standard infant feeding indicators are comprehensive and the adjustments for sampling design made in the analysis are appropriate. The DHS datasets used in the analyses are nationally-representative in design. A common survey methodology was applied to support comparison with DHS datasets of other countries. A limitation of this study was that the EISF was based on a self-reported, 24-h recall rather than the situation over the entire period from birth. This is the method recommended by the WHO for national surveys and international comparisons [36] but could have resulted in a potential change in classification of infants fed with pre-lacteals or other foods prior to the 24 h. Other limitations were that the survey considered the timeliness of introduction of formula and/or solid, semi-solid of soft foods but not on the quantity or frequency of foods given and did not collect information on the provision of formula (non-human milk).

## 5. Conclusions

This study revealed factors significantly associated with early introduction of formula and/or solid, semi-solid or soft foods among infants aged three to five months across seven Francophone West African countries. These factors included: acute respiratory infection and diarrhoea contracted by infants (Benin, Guinea and Senegal), male gender (Cote d’Ivoire and Senegal), infants in urban areas (Senegal), and infants delivered by traditional birth attendants (Guinea, Niger and Senegal).

Successful interventions to discourage early introduction of complementary feeding need to focus on those factors by targeting these specific groups in health promotion programmes, in healthcare delivery, and as an important focus for health worker and TBA training and education.
